# Characterization of *Yersinia pestis* Phage Lytic Activity in Human Whole Blood for the Selection of Efficient Therapeutic Phages

**DOI:** 10.3390/v13010089

**Published:** 2021-01-11

**Authors:** Sarit Moses, Yaron Vagima, Avital Tidhar, Moshe Aftalion, Emanuelle Mamroud, Shahar Rotem, Ida Steinberger-Levy

**Affiliations:** Department of Biochemistry and Molecular Genetics, Israel Institute for Biological Research, Ness-Ziona 74100, Israel; Sarit5761@gmail.com (S.M.); yaronv@iibr.gov.il (Y.V.); avitalt@iibr.gov.il (A.T.); moshea@iibr.gov.il (M.A.); emmym@iibr.gov.il (E.M.); Shaharr@iibr.gov.il (S.R.)

**Keywords:** bacteriophage, phage selection, personalized phage therapy, human whole blood, *Yersinia pestis*

## Abstract

The global increase in multidrug-resistant (MDR) pathogenic bacteria has led to growing interest in bacteriophage (“phage”) therapy. Therapeutic phages are usually selected based on their ability to infect and lyse target bacteria, using in vitro assays. In these assays, phage infection is determined using target bacteria grown in standard commercial rich media, while evaluation of the actual therapeutic activity requires the presence of human blood. In the present work, we characterized the ability of two different *Yersinia pestis* lytic phages (ϕA1122 and PST) to infect and kill a luminescent *Y. pestis* EV76 strain suspended in Brain Heart Infusion (BHI)-rich medium or in human whole blood, simulating the host environment. We found that the ability of the phages to infect and lyse blood-suspended *Y. pestis* was not correlated with their ability to infect and lyse BHI-suspended bacteria. While the two different phages exhibited efficient infective capacity in a BHI-suspended culture, only the PST phage showed efficient lysis ability against blood-suspended bacteria. Therefore, we recommend that for personalized phage therapy, selection of phage(s) for efficient treatment of patients suffering from MDR bacterial infections should include prior testing of the candidate phage(s) for their lysis ability in the presence of human blood.

## 1. Introduction

Pandemics, either viral-derived, such as the recently emerged COVID-19, or bacterial-derived, such as reemerging plague, are of major public health concern. Although antibiotic administration is a common and efficient approach to fight bacterial-derived infections, there has been a global spread of multidrug-resistant (MDR) bacteria, leading to high mortality rates among bacterial-infected patients [1,2,3]. The spread of MDR bacteria is the result of human and livestock antibiotic misuse, leading to selective pressure toward microbial resistance. Since the introduction of penicillin in the 1940s, bacterial resistance has evolved rapidly to each novel antibiotic that has entered the drug market. As a result, drug companies have decreased their investments and efforts in the discovery, design and development of new antibiotic compounds. Recently, the World Health Organization (WHO) published two reviews describing the worldwide status of preclinical and clinical antibacterial development, emphasizing the decline in initiatives to reduce antimicrobial resistance [4,5]. Therefore, an alternative antimicrobial therapy is urgently needed. Lytic bacteriophages may serve as a medical tool for the treatment of MDR pathogens.

Bacteriophages (phages) are environmentally abundant viruses that specifically invade and replicate inside host bacteria, forming novel progenitor virions that lyse the bacteria from within and enable the spread of the viruses for the next infection cycle. Since their independent discovery by Frederik Twort (in 1915) and Felix d’Herelle (in 1917) [6], isolated phages have been used for the treatment of various infectious diseases, including dysentery, cholera and plague [6,7,8]. However, since the appearance of antibiotics in the 1940s, phage usage for therapy was disregarded in Western countries, although it continued to serve as an accepted therapy method in the former USSR, Georgia and Eastern European countries, such as Poland [8]. Recently, because of the global emergence of MDR bacteria, phage therapy has been largely investigated in Western countries as well as a renewed approach for the treatment of infectious diseases. Research has been conducted using animal models of various infectious diseases treated with phages alone [9,10] or in combination with antibiotics [11,12,13]. Moreover, preclinical and clinical trials have been conducted against various human pathogens [14,15]. Recently published case reports described the successful treatment of some MDR-infected patients by administration of antibiotics in combination with a cocktail of phages personally selected to lyse the patient’s isolated pathogen [16,17,18,19,20].

*Yersinia pestis* is the causative agent of plague, a lethal infectious disease that has caused several pandemics throughout human history, claiming the lives of millions of people [21,22]. Plague is recognized as a reemerging disease, and the most recent large outbreak took place in Madagascar in 2017, where many of the patients had pneumonic plague [23]. Due to its lethality and high infectivity, *Y. pestis* is classified by the Centers for Disease Control and Prevention (CDC) as a tier-1 select bioterrorism agent [21,24]. The most prevalent form of the disease in nature is bubonic plague, which is acquired through fleabites, and without effective treatment, it can also spread into the lung, leading to a pneumonic disease [25]. The pneumonic form of the disease is fatal if not treated with proper antibiotics on time [22]. Most wild-type *Y. pestis* strains are sensitive to the recommended antibiotics; however, some antibiotic-resistant strains have been isolated from humans and rodents [26,27,28]. Thus, in the case of MDR *Y. pestis*, phage therapy may be considered an important alternative therapy.

*Y. pestis* infection, similar to other bacterial infections, can develop into a systemic disease [25], and as such, for the treatment of antibiotic-resistant bacteria, it is crucial to select a therapeutic phage that is able to lyse the pathogen while it is circulating in the blood or exists in “bloody” tissues (such as the spleen and liver). It was shown that the activity of some phages is inhibited in the presence of human blood [29,30]. Thus, we assessed here the ability of two different *Y. pestis*-lysing phages, ϕA1122 and PST, to infect and kill their host bacteria in the presence of human blood.

## 2. Materials and Methods

### 2.1. Bacterial Strains, Bacteriophages and Growth Media

The bacterial strains used in this study were the *Y. pestis* EV76 live vaccine strain (accession no. PRJNA647169) and its bioluminescence recombinant derivate EV76::*lux* containing the *luxCDABE* cassette [31]. Nonvirulent *Y. pestis* Kimberley53∆70∆10 [32] served as a host for phage lysate preparation and phage titration. Bacteria were grown on Brain Heart Infusion Agar (BHIA) and suspended in BHI liquid growth media (purchased from Bactlab Diagnostics Ltd., cat. nos. 241830 and 237500, respectively).

The *Y. pestis*-specific lytic bacteriophages used in this study were ϕA1122 (accession no. NC004777, kindly provided by Prof. Mikael Skurnik [33,34]) and PST (ATCC, cat. no.: 23207-B1; accession no. KF208315).

### 2.2. Human Blood

Human whole-blood donations (using CPDA1 as an anticoagulant) were provided by the Israeli Blood Bank and were kept at 4 °C until use. The blood samples were used within 2 weeks of uptake.

### 2.3. Bacteriophage Preparation

Bacteriophage lysates were prepared by the liquid lysis protocol [35]. Briefly, *Y. pestis* Kimberley53∆70∆10 culture was grown at 37 °C and 200 rpm to an optical density at 660 nm (OD_660 nm_) = 1.5. Phages were added at a multiplicity of infection (MOI) = 0.001, and incubation was continued for another 6 h for ϕA1122 phage or overnight (ON) for PST phage, to achieve bacterial lysis. Phage supernatants were clarified by centrifugation at 2900× *g* for 10 min at room temperature (RT), followed by 0.2 µm filtration and storage at 4 °C in the dark until use.

### 2.4. Bacteriophage Titration

The phage titer was determined by plaque assay or spot assay using the agar overlay method [35]. For the spot assay, a bacterial lawn was prepared by mixing 0.3 mL of logarithmic *Y. pestis* Kimberley53∆70∆10 culture (grown in BHI at 200 rpm to OD_660 nm_ = 0.5) with 2.7 mL of 0.6% or 0.4% (for ϕA1122 or PST, respectively) molten top BHIA (50 °C), poured on a 20 mL BHIA plate (bottom 1.5% agar) and incubated for 20 min at RT. Triplicates of 10 µL of the phage 10-fold serial dilutions in SM buffer (0.1 M NaCl, 8 mM MgSO_4_, 50 mM Tris-HCl pH 7.5 and 0.01% Gelatin solution) were spotted on the top agar. The spots were allowed to fully adsorb onto the top agar, followed by ON incubation at RT for ϕA1122 or at 37 °C for PST. Plaques were counted the next day.

### 2.5. Absorbance- and Bioluminescence-Based Lysis Assay

The EV76 and EV76::*lux* strains were grown on BHIA at 37 °C for 48 h. Bacterial colonies were suspended in PBS and inoculated (1:10; vol:vol) in BHI broth or in human whole blood and transferred (90 µL/well) into a 96-well transparent-bottom white microplate (Thermo Scientific Nunc: cat. no. 165306). Infection was performed by adding 10 µL of phage solution (MOI = 0.01) or 10 µL of SM buffer to the growth control wells. The bacterial growth curves were assessed by tracking the OD_630nm_ or the bioluminescent signal (relative light units, RLU) of each well at 15-min intervals over 24 h using a SPARK 10 M plate reader (Tecan). The temperature in all experiments was 37 °C.

### 2.6. Phage and Bacterial Propagation in BHI and in Human Blood

Prewarmed (37 °C) BHI or human blood was spiked with 10^6^ CFU/mL *Y. pestis* strain EV76. For the adjustment of the bacteria to the blood environment, cultures were incubated for 1 h at 37 °C and 150 rpm. For phage infection, the BHI- or blood-grown cultures were divided into 3 conical 50-mL tubes (5 mL in each tube) and infected separately with *Y. pestis*-specific bacteriophages ϕA1122 or PST (50 µL) at an MOI = 0.01 (1 × 10^4^ PFU/mL). Growth control cultures were not infected by the phages. For time 0 phage titration, 50 µL of phage solution was added to 5 mL SM buffer, followed by serial 10-fold dilutions, as performed for the other samples. The cultures were incubated at 37 °C and 150 rpm for 24 h, and samples (0.5 mL) were taken for phage titration and bacterial live counting at 0, 6, 12 and 24 h post infection. For live counting, samples were serially diluted 10-fold in PBS, and 10 µL of the diluted bacteria was spotted in triplicate on BHIA and incubated at 28 °C for 48 h. For phage titration, separation of phages from cellular fraction was done by centrifugation in Eppendorf tubes at 1700× *g* (in order to avoid blood cell hemolysis), for 5 min at RT. The supernatants were serially diluted 10-fold in SM buffer and used for phage titration as described above.

### 2.7. Heat Inactivation of Human Whole Blood

Blood was centrifuged at 2000× *g* for 10 min at RT. Supernatant plasma was transferred to another tube and incubated at 56 °C for 60 min and then gently mixed back with the blood cellular fraction.

## 3. Results

### 3.1. Comparing Phage Lytic Efficiency of Broth-Suspended Y. pestis Culture

**Figure 1:**
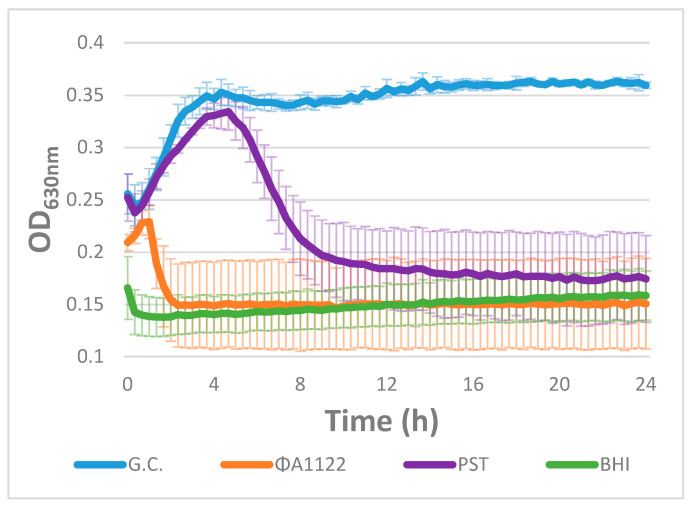
Lysis curve of EV76 cultures infected by phages. A *Y. pestis* EV76 bacterial suspension (90 µL of 10^8^ CFU/mL) divided in a 96-well microplate was infected with two different *Y. pestis*-specific phages (ϕA1122 and PST; 10 µL of 10^7^ PFU/mL) at an MOI of 0.01 (time 0 is time of infection). Growth/lysis curves were assessed by tracking the OD_630nm_ at 37 °C for 24 h in 15-min intervals using a Spark 10 M plate reader. The results are representative of one of three independent biological replicates. Values are the average of four replicate wells in the same experiment, and the error bars represent the standard deviation (STDEV). G.C. = Growth Control.

The selection of therapeutic lytic phages for humans is performed by comparing the in vitro lysis efficiencies of various available relevant phages [16,17,18,19,20,36]. Phage lysis efficiency can be assessed either on semisolid agar plates by plaque assay, spot assay or efficiency of plating (EOP) or by the liquid-based lysis test, which follows the reduction in the OD of broth-suspended bacterial culture [37]. Here, we compared the lysis efficiency of two different *Y. pestis* phages, ϕA1122 and PST [33], for their ability to lyse and thus reduce the OD of *Y. pestis* liquid culture. As seen in Figure 1, ϕA1122 showed higher lytic activity, as it lysed the bacteria completely within 2.5 h post infection. Complete lysis by the PST phage was achieved only at 10 h post infection.

Since lysis of broth-suspended bacteria is considered a good tool for the selection of therapeutic phages, ϕA1122 would be the ultimate candidate for phage therapy in patients infected with MDR *Y. pestis*. Nevertheless, plague can develop into a bacteremic/systemic disease [25], meaning that the selected phage(s) must also be infective in the blood environment and not only in broth media. Since some phages are inactive in the presence of blood [29,30], we conducted lysis assays in the presence of human blood as well.

### 3.2. Comparing Phage Lytic Activity in Human Whole Blood vs. Broth Laboratory-Rich Medium

To track bacterial growth and lysis kinetic in the presence of blood, which quenches OD measurement, we looked for an alternative method. Since a bioluminescent signal is readable even in the presence of blood components, we inserted the *luxCDABE* operon cassette (encoding the Luciferase enzyme and its substrate) into *Y. pestis* EV76 and used the bioluminescent-derived strain, EV76::*lux*, as the host bacteria. The EV76::*lux* growth kinetic profile observed by monitoring the OD resembled that detected by monitoring bioluminescence (Supplementary Materials Figure S1).

**Figure 2:**
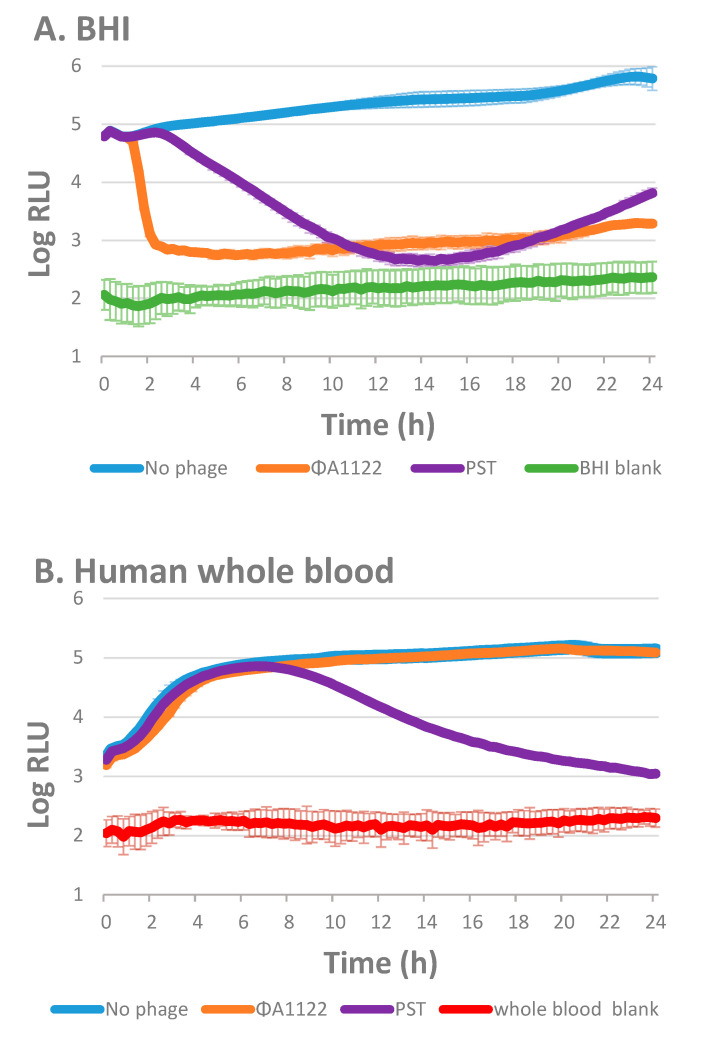
Phage lysis of EV76::*lux* suspended in Brain Heart Infusion (BHI) or in whole blood. A bioluminescent lysis assay was performed with 90 µL of 10^7^ CFU/mL EV76::*lux* suspended in BHI (**A**) or in human whole blood (**B**) and infected with 10 µL of ϕA1122 or PST phages (10^6^ PFU/mL; MOI = 0.01). Bioluminescence was assessed by tracking the relative light units (RLU) at 37 °C in 15-min intervals for 24 h using a Spark 10 M plate reader. The experiment was performed using 10 different blood donors (of various blood types) and the results are representative of one blood donor (blood type O+, 9 days from uptake). Values are the average results from 3 wells triplicate in the same experiment, and the error bars represent the standard deviation (STDEV).

We compared the bioluminescence curves of the two phage-infected bacterial cultures in the presence of broth medium ([Brain Heart Infusion BHI], Figure 2A) or human blood (Figure 2B). As seen in the absorbance-based lysis assay of EV76 (Figure 1), the bioluminescent tracking assay revealed that phage ϕA1122 is the most efficient lysing phage for the BHI-suspended EV76::*lux* culture, being the fastest out of the two tested phages to reduce bacterial bioluminescence to the background level (Figure 2A). PST also behaved similarly as in the absorbance-based lysis assay (as shown in Figure 1) and lysed the culture within 12 h.

In contrast to the lysis profile seen in the BHI-suspended culture, in the presence of human blood, the phage lysis profiles changed, and the lysis level was reduced or totally inhibited, depending on the tested phage (Figure 2B). Phage ϕA1122 did not show any lytic activity, while the PST phage could lyse *Y. pestis* in the presence of human blood (Figure 2B), although at a slower rate than the broth-suspended bacteria.

ϕA1122 and PST both bind to the *Y. pestis* lipopolysaccharide (LPS) molecule, which serves as their receptor [33]. However, each phage binds to a different region of the LPS molecule [33]. Thus, we assumed that the difference in the observed blood inhibitory effect may occur via interference with phage–receptor interactions by some blood components, such as complement. Therefore, we tested whether heat inactivation, which inhibits complement activity [30], changed the blood inhibition of phage lytic activity.

### 3.3. The Effect of Blood Heat Inactivation on Blood Inhibition of Phage Lysis

**Figure 3:**
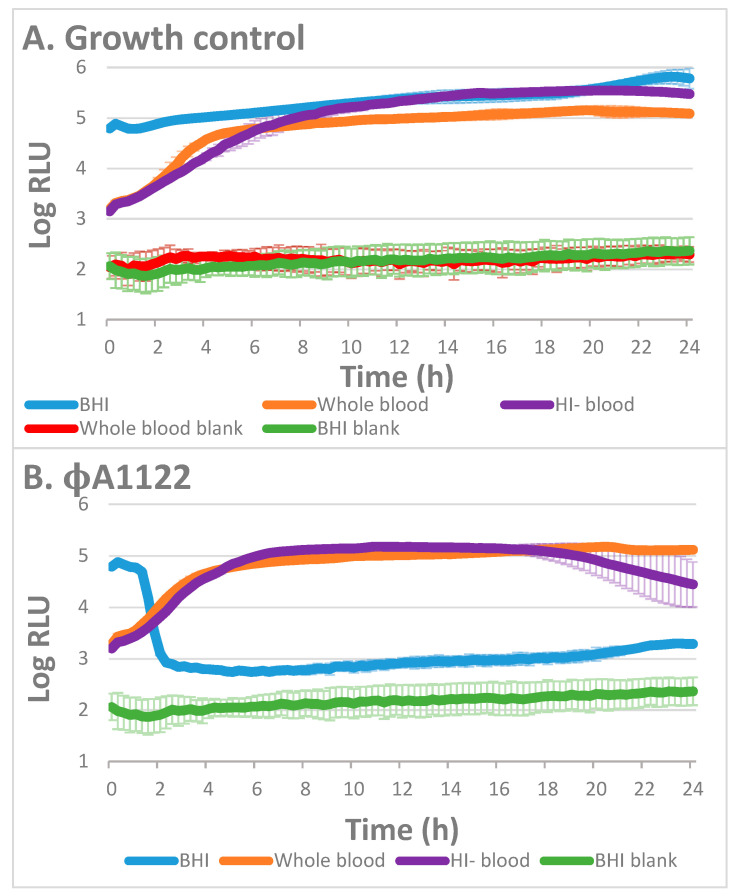

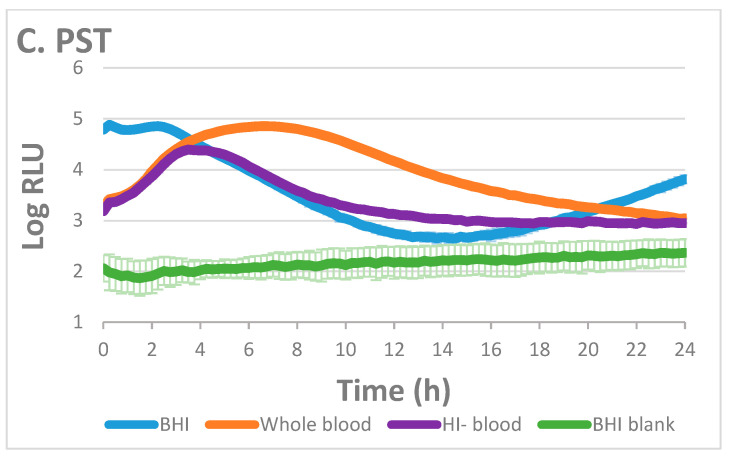
Lysis of EV76::*lux* in BHI, whole blood and HI-blood by *Y. pestis* phages. A bioluminescence lysis assay was performed with 10^7^ CFU/mL EV76::*lux* spiked in BHI, human whole blood or HI-blood. The bioluminescence assays were assessed without phage (**A**) or with ϕA1122 (**B**) or PST phage (**C**) at MOI = 0.01. The experiment was performed using 6 different blood donors (of various blood types), and the results are representative of one blood donor (blood type O+, 9 days from uptake). Values are the average relative light units (RLU) results from 3 wells triplicate in the same experiment, and the error bars represent the standard deviation (STDEV).

For blood heat inactivation, we separated the plasma from the cellular fraction, heated it for 1 h at 56 °C and returned the heat-inactivated plasma to the cellular fraction (herein, HI-blood). As depicted in Figure 3A, bacterial growth in the presence of HI-blood was similar to that observed in the presence of blood. Interestingly, we found that the heat inactivation influenced the lytic activity of the phages in a different way (Figure 3). While phage ϕA1122 was similarly inhibited in the presence of blood or HI-blood (Figure 3B), PST lytic activity was enhanced in the presence of HI-blood (Figure 3C), suggesting that the complement slightly interferes with its lytic activity.

### 3.4. Monitoring Phage Titer Increments in Human Blood-Suspended Bacteria as a Tool for Customized Phage Selection

For the selection of optimal phages that are capable of lysing the bacteria in the presence of the blood, we suggest conducting a simple phage titration assay. This assay is carried out by incubating the patients’ blood sample inoculated with the patients’ isolated pathogenic bacteria and infected by the phage(s) in question. Phage replication is determined by titration at the time of infection (time = 0) and following several hours of incubation. An increase in phage titer indicates the killing of the tested bacteria by the phages. Comparing the titer increment level of various tested phages enables the selection of the most efficient phage(s) for the treatment of systemic-derived infectious bacteria.

**Figure 4:**
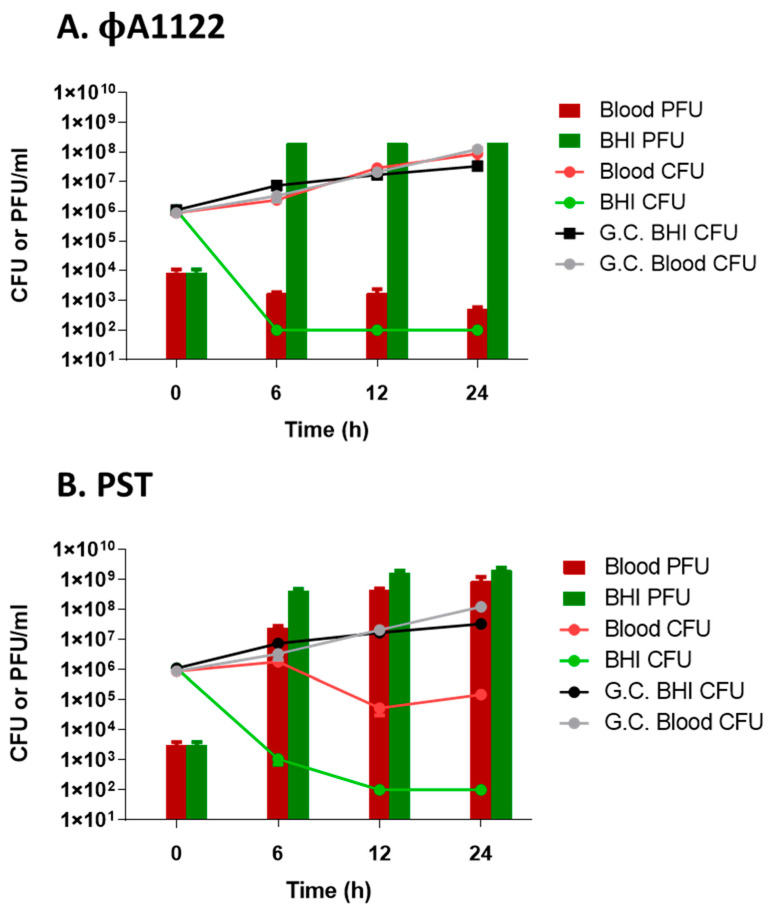
Phage and bacterial propagation assay. First, 37 °C-prewarmed BHI or human blood (blood type O+, 6 days from uptake) was spiked with 10^6^ CFU/mL *Y. pestis* strain EV76 and incubated at 37 °C and 100 rpm for 1 h followed by ϕA1122 (**A**) or PST phage (**B**) infection (MOI = 0.01). The infected cultures were incubated at 37 °C and 150 rpm for 24 h. Samples (0.5 mL) were taken at the time of infection (T = 0) and 6, 12 and 24 h post infection. For bacterial enumeration, samples were serially diluted 10-fold in PBS, and 10 µL of the diluted bacteria was spotted in triplicate on BHIA and incubated at 28 °C for 48 h. For phage titration, samples were centrifuged at 1700× *g* for 5 min at RT, and the supernatants were serially diluted 10-fold in SM buffer and used for phage titration by spot assay. Values are the average of triplicate spots in the same experiment, and the error bars represent the standard deviation (STDEV). G.C.= Growth Control.

Here, we tracked the titer increment of two *Y. pestis*-infecting phages over 24 h post infection of *Y. pestis* bacteria suspended in BHI or in human blood. We found that both tested phages were infective in BHI-suspended bacteria, as phage titers at time = 6 h were increased by ~5 orders of magnitude (Figure 4). These phages were already proven by both absorbance and bioluminescence lysis assays to be efficient lysing phages of BHI-suspended *Y. pestis* culture (Figure 1 and Figure 2A). In comparison to that of the BHI-suspended culture, the phage titer of the 6 h post infection blood-suspended culture showed a difference in phage propagation ability (Figure 4). ϕA1122 was totally inhibited in the presence of blood, with a reduction of 5 orders of magnitude in the blood-derived phage titer compared to the BHI-derived phage titer. In contrast, the PST phage titer increased 4 orders of magnitude at time = 6 h, showing a minor reduction of only 1 order of magnitude compared to the 6-h BHI-derived titer (Figure 4B). Moreover, prolonged incubation did not improve ϕA1122 propagation, while PST showed a further increase in phage titer, similar to the BHI-derived phage titer. Inhibition of ϕA1122 propagation in the presence of blood correlates with bacterial propagation in the blood, where bacterial live counting results resembled the growth control counting results (Figure 4A), suggesting that in the blood environment, ϕA1122 could not lyse its target bacteria. In contrast, the ability of PST to propagate in the blood correlates with a reduction in bacterial counting (Figure 4B), emphasizing its ability to lyse *Y. pestis* in the blood environment.

In summary, out of the two tested lytic phages, ϕA1122 showed the best lytic activity of broth-suspended *Y. pestis*. However, this phage was totally inhibited in the presence of human blood. In contrast, the PST phage showed higher potential for efficient lysis of *Y. pestis* in the presence of human blood and thus may be a better candidate for phage therapy in the case of plague caused by antibiotic-resistant *Y. pestis* strains.

## 4. Discussion

Phage therapy may serve as an alternative treatment method to combat the worldwide increase in MDR pathogens. In addition to natural selection of the MDR *Y. pestis* strain, there is a bioterrorism concern regarding the deliberate selection of MDR bacteria. Thus, in the case of MDR *Y. pestis*, phage therapy may be considered an alternative therapy. The potential of such a treatment was suggested almost a century ago in studies by Felix d’Herrele, who treated patients suffering from bubonic plague using *Y. pestis*-specific phages [33].

The selection of phages for the preparation of a therapeutic phage cocktail is crucial for successful treatment, as phages can differ from each other by their lytic efficiency, host range, synergism with antibiotics, etc. Recently published case reports described the preparation of a therapeutic phage cocktail using the patient’s isolated pathogenic bacteria as a host for the selection of candidate virulent phages. Phages were selected from phage collection or from environmental samples by direct or indirect in vitro assays, such as spot assays, plaque assays and liquid-based lysis assays [16,17,18,19,20]. These assays determine the ability of the candidate phages to lyse and kill the patient’s isolated bacteria when suspended in rich growth media [37].

Here, we conducted a lysis test using two different phages, ϕA1122 and PST, to identify an efficient lytic *Y. pestis* phage. Lysis tests in broth-suspended bacteria revealed that phage ϕA1122 has the most rapid lysing ability and can kill *Y. pestis* within a very short time (Figure 1). Previous reports showed that ϕA1122 is a universal phage and can lyse almost all tested *Y. pestis* strains [38] and thus it has been used by the CDC for *Y. pestis* diagnostics [39]. Moreover, the appearance of bacterial resistance to ϕA1122 is very rare since its receptor, the LPS molecule, is a critical virulence factor; thus, LPS mutants of *Y. pestis* are strongly attenuated [33]. Therefore, ϕA1122 seems to be the optimal therapeutic phage for plague disease. However, since *Y. pestis* may enter the circulatory system and produce systemic disease, and since ϕA1122 activity is evaluated in standard laboratory growth media, we tested the lytic efficiency of the phages in the presence of human blood. We found that ϕA1122 lytic activity was abolished in a human blood environment (Figure 2B and Figure 3B), suggesting that it is not the best phage candidate for the treatment of plague infection. In contrast, PST was able to efficiently infect *Y. pestis* bacterial cells in the presence of blood, indicating that it may serve as an attractive candidate for antiplague phage therapy (Figure 2B). This phage-dependent inhibitory effect may suggest that blood-derived factor(s) affect phage lytic activity, in a way that interferes with phage–bacterial interactions.

The observed reduction in *Y. pestis* lysis by ϕA1122 phage and not by PST phage, in the blood environment, may be explained by several mechanisms, which are as follows. 1—Interference with phage adsorption and binding to its specific bacterial receptor. The receptor for both ϕA1122 and PST is the LPS molecule [33]. However, while ϕA1122 binds to the inner core of LPS (specifically to the Kdo/Ko moiety in the LPS molecule [33,34]), PST binds to the outer core of LPS (Hep(II)/Hep(III) of LPS) [33]. Thus, some small blood component(s) may bind to the bacterial surface, interfering with the interaction of ϕA1122 with the inner core of LPS but not with the binding of PST to the outer core of LPS. 2—Soluble factors in the blood may scavenge phages and the major differences (including phage structure, size, capsid protein composition, etc.) between the T7-like phage, ϕA1122, and PST, which is a T4-like phage [33], may differentially affect phage lytic activity. 3—Exposure of *Y. pestis* to the human blood environment alters gene expression, resulting in changes in membrane and cellular protein composition [40]. This may differentially influence phage propagation within the bacteria as well as its lytic activity.

Notably, identifying the bacterial receptor targeted by therapeutic candidate phages can facilitate the selection of efficient phages for the preparation of phage cocktails, in which phages bind to different receptors. Such a cocktail will reduce the possible development of phage-resistant bacteria and increase the chances of targeting a receptor that is not inhibited by the blood.

Aiming to determine whether binding of the complement molecule to the bacterial surface may interfere with phage infection, we heat-inactivated the blood (HI-blood) prior to bacterial inoculation followed by phage infection. We found that for the PST phage, heat inactivation neutralized the minor blood inhibition of its lytic activity (Figure 3C), suggesting that the complement molecule slightly interferes with PST binding to its receptor. In contrast to PST phage, heat inactivation did not neutralize the blood inhibitory effect observed for ϕA1122 (Figure 3B), suggesting that the complement molecule is not involved in the blood inhibition of its lytic activity and that other/additional blood components may be involved. Currently, the mechanism of the inhibitory effect of human blood on ϕA1122 activity is unknown. Further study of various phages (targeting various phage receptors), bacterial hosts and human blood of different origins (different types and donors), as well as the addition of purified blood components, may allow a better understanding of the mechanism that underlies the blood inhibitory effect on phage lytic activity and explain the cause for the different effects observed for different phages. Moreover, searching for additional MDR *Y. pestis*-targeting lytic phages in the presence of blood is required, aiming to establish an efficient phage cocktail.

As we show here, the activity of a phage in laboratory growth medium does not indicate whether it will be active in blood. Therefore, a preliminary test of phage lytic activity in the presence of human blood should be conducted before the administration of the candidate phage(s) to a patient, especially when treating an infectious systemic (or potentially systemic) disease. It should be mentioned that for severe and fast progressive disease, such as the plague disease, characterization of phage activity in human blood (of various blood types) should be performed prior to disease outbreak. However, in the case of chronic or slow progressive disease, where candidate phages are personally selected to lyse the patients’ isolated MDR bacteria, a lysis test should be done in the presence of the patients’ blood sample.

For this purpose, we suggest that following the selection of candidate phages via lysis assays in growth medium, a simple phage titration assay should be performed in the presence of the patient’s blood. Comparison of different phage propagation abilities will allow the selection of a phage that has the best lytic activity in the presence of patient’s blood. Moreover, as complement interferes with only some receptors, comparison of the effect of HI-blood on different candidate phages may differentiate between phages targeting different bacterial receptors and allow the targeting of various receptors by a phage cocktail. The titration assay required several hours for the phage propagation step (we tested phage propagation 24 h post infection and already observed differences in phage efficiencies 6 h post infection; Figure 4) and an additional several hours of ON incubation for the plaque assay for phage titration. Thus, the overall assay is rapid, and a proper therapeutic phage may be chosen within 24 h of the assay.

In summary, in the present work, we show that phage lytic activity may be influenced by the presence of human whole blood. Therefore, we recommend that for personalized phage therapy, a selection step performed to identify the best therapeutic phage candidates should include testing phage lytic activity in the presence of the patient’s blood, especially when treating a systemic infectious disease.

## Data Availability

Not relevant.

## Supplementary Materials

The following are available online at https://www.mdpi.com/1999-4915/13/1/89/s1, Figure S1: Comparison of the bioluminescence and absorbance growth curves of EV76::*lux*.
