# Supplementary material for: Characterization of Yersinia pestis Phage Lytic Activity in Human Whole Blood for the Selection of Efficient Therapeutic Phages
Source: Viruses. 2021 Jan 11;13(1):89. doi: 10.3390/v13010089 (PMC7827537; doi:10.3390/v13010089)
Supplement: Supplementary file 1 [file viruses-13-00089-s001.pdf]

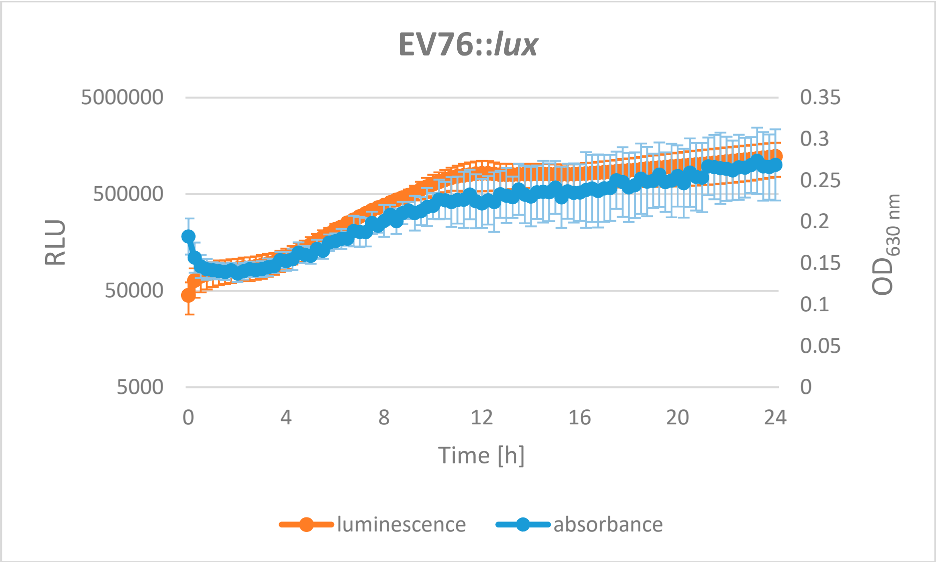

Figure S1: Comparison of the bioluminescence and absorbance growth curves of EV76::*lux*. EV76::*lux* colonies grown on BHIA at 37°C were suspended in BHI (10^8 CFU/ml) and divided (100 µl/well) into 6 wells in a 96-well white microplate with a transparent bottom. Growth curves were assessed by tracking $OD_{630\ nm}$ and bioluminescence (RLU) at 37°C for 24 h in 15 min intervals using a Spark 10M plate reader. Values are the average of six replicate wells, and the error bars represent the STDEV.
